# Unexpected high carbon losses in a continental glacier foreland on the Tibetan Plateau

**DOI:** 10.1038/s43705-022-00148-x

**Published:** 2022-08-09

**Authors:** Jiejie Zhang, Anzhou Ma, Hanchang Zhou, Xianke Chen, Xiaorong Zhou, Guohua Liu, Xuliang Zhuang, Xiang Qin, Anders Priemé, Guoqiang Zhuang

**Affiliations:** 1 Research Centre for Eco-Environmental Sciences, Chinese Academy of Sciences, Beijing, 100085 China; 2 Sino-Danish College of University of Chinese Academy of Sciences, Beijing, 101400 China; 3 Sino-Danish Center for Education and Research, Beijing, 101400 China; 4 College of Resources and Environment, University of Chinese Academy of Sciences, Beijing, 100049 China; 5 Institute of Tibetan Plateau Research, Chinese Academy of Sciences, Beijing, 100101 China; 6 Qilian Shan Station of Glaciology and Eco-environment, State Key Laboratory of Cryospheric Science, Northwest Institute of Eco-environment and Resources, Chinese Academy of Sciences, Lanzhou, 730000 China; 7 Department of Biology, University of Copenhagen, Copenhagen, DK-2100 Denmark; 8 Center for Permafrost, University of Copenhagen, Copenhagen, DK-1350 Denmark

**Keywords:** Microbial ecology, Biogeochemistry, Climate-change ecology

## Abstract

Closely related with microbial activities, soil developments along the glacier forelands are generally considered a carbon sink; however, those of continental glacier forelands remain unclear. Continental glaciers are characterized by dry conditions and low temperature that limit microbial growth. We investigated the carbon characteristics along a chronosequence of the Laohugou Glacier No. 12 foreland, a typical continental glacier on the Tibetan Plateau, by analyzing soil bacterial community structure and microbial carbon-related functional potentials. We found an unexpected carbon loss in which soil organic carbon decreased from 22.21 g kg^−1^ to 10.77 g kg^−1^ after receding 50 years. Structural equation modeling verified the important positive impacts from bacterial community. Lower carbon fixation efficiency along the chronosequence was supported by less autotrophic bacteria and carbon fixation genes relating to the reductive tricarboxylic acid cycle. Lower carbon availability and higher carbon requirements were identified by an increasing bacterial copy number and a shift of the dominant bacterial community from Proteobacteria and Bacteroidetes (*r*-strategists) to Actinobacteria and Acidobacteria (K-strategists). Our findings show that the carbon loss of continental glacier foreland was significantly affected by the changes of bacterial community, and can help to avoid overestimating the carbon sink characteristics of glacier forelands in climate models.

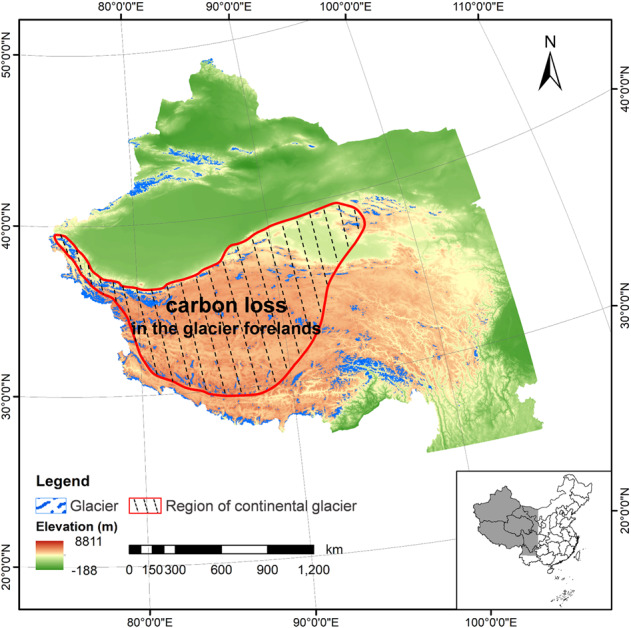

## Introduction

Accelerated glacier retreat due to global warming, with a global temperature rise of 1.09 °C in recent years, has led to increases in exposed soils [1]. These glacier forelands are characterized by limited area and a harsh environment with low temperature and poor soil nutrient contents [2], especially that of carbon, the basic element controlling soil development [2–4]. In general, the accumulation rate of soil organic carbon (SOC) is the greatest in the initial soil development stage, compared with subsequent soil development in glacier forelands [5, 6], accompanied by accumulated carbon resources [7]. This increase of carbon in glacier forelands has been observed in the Italian north-western Alps [8], Norway [9], SE Iceland [10], Antarctic Peninsula [11], and the High Arctic [12].

Regional variations in original carbon resources, climatic factors, and edaphic factors significantly impact the carbon accumulation in glacier forelands [2, 13, 14]. The foreland of Hailuogou Glacier, a typical temperate glacier on the Tibetan Plateau, developed into a coniferous forest with an SOC accumulation rate of 14 g m^−2^ year^−1^ between 1890 and 2010 [15, 16]. In contrast, the total carbon of foreland soils in the Urumqi Glacier No. 1 (the sub-continental glacier) only increased by approximately 1.35 g kg^−1^ after 44 years of succession [17, 18]. To date, there have been no related studies on Chinese continental glaciers, which account for an area of 19,000 km^2^, one-third of the total glacial area in China [19]. Continental glaciers are characterized by low annual average temperature (below 0 °C) and very dry conditions with an annual precipitation of 200~500 mm. They are sensitive to climate change, with significant receding having been recorded [20]. For example, Laohugou Glacier No. 12 decreased in area by 1.54 km^2^ between 1957 and 2015 [21]. It is therefore necessary to study the carbon characteristics of continental glacier forelands to obtain a more comprehensive understanding of glacier retreat and its feedback effects on global warming.

In addition to external inputs such as supraglacial input and aerial deposition, biological activities are the major source of nutrients in the glacier forelands [3, 22]. Before vegetation sucession can begin, carbon accumulation mainly depends on microorganisms, especially bacteria, which are the major pioneer colonizers in glacier forelands [23–27]. Therefore, the balance of microbial metabolic respiration and carbon fixation would greatly influence carbon storage in glacier forelands [28–30]. As exposure time increases and the environment improves, the microbial metabolism including fixation and consumption would be strengthened [2, 31, 32]. Moreover, these shifts are also closely related to the change of microbial community composition such as the relative proportions of autotrophs and heterotrophs [9, 33] and the abundance of functional genes [34, 35]. Functional genes can not only suggest the possible existence of corresponding metabolic pathways and their microbial functional potentials [35, 36], but can also greatly improve our understanding of nutrient cycling, which is mediated by microorganisms [37].

To improve the current understanding of carbon accumulation in continental glacier forelands, we focused on the dynamics of carbon mediated by bacteria along the soil chronosequence in the continental glacier forelands by analyzing successional characteristics of microbial functional potentials and microbial community structure based on soil samples from the typical representative of continental glacier foreland, Laohugou Glacier No. 12. We hypothesized that despite the specific dry and low temperature conditions, carbon would accumulate along the continental glacier forelands, similar to other types of glacier forelands, which is closely related to changes in the microbial community.

## Materials and methods

### Study sites and soil sampling

Laohugou Glacier No. 12, the largest continental glacier in the Tibetan Plateau, is located on the northern slope of the western Qilian Mountains, at 39°26.4′N, 96°32.5′E with an elevation of 5483 m including two attributes [21]. Its annual average temperature is −11.8 °C with typical continental climate in which the precipitation (about 390 mm year^−1^) is mainly concentrated in late summer to early autumn and the prevailing wind is westerly [38, 39].

**Fig. 1:**
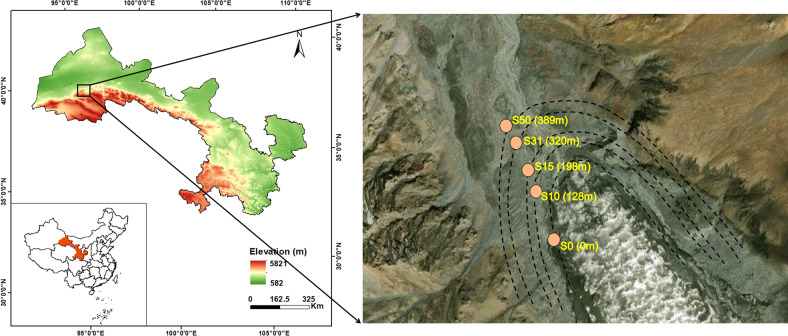
Location of Laohugou Glacier No. 12 within China and the position of the sampling sites in the glacier foreland. Numbers in the five sampling sites represent the retreat years (S0, S10, S15, S31, and S50). Numbers in brackets represent their distances from the ice tongue.

Under the influence of global warming, the terminus of Laohugou Glacier No. 12 retreated 403 m from 1960 to 2015, equivalent to 7.3 m year^−1^ [21, 40]. Based on this retreat rate, five sampling sites were positioned by the distance from the terminus corresponding to a recession after 0, 10, 15, 31, and 50 years (denoted S0, S10, S15, S31, and S50, respectively) (Fig. 1 and Table S1). No plants were found at S0, S10, and S15, while S31 and S50 were extremely sparely vegetated. *Thylacospermum caespitosum* and *Ajania scharnhorstii* were found at S31 and S50, respectively, where they were distributed in patches. In November 2020, surface soil samples were obtained in triplicate from each site. After removing the large particles and sieving at 2 mm, each sample was divided into two parts (one for soil physicochemical property analysis and the other for microbial molecular analysis) and then transported to the laboratory at 4 °C.

### Analysis of soil physicochemical properties

The slurry method was applied for soil pH measurement, wherein 2 g of air-dried soil was mixed with 10 mL of water (soil: water [m/v] = 1:5) and left to stand for 30 min after shaking for 2 min, and the supernatant was analyzed for pH with an electrode. The water content was measured gravimetrically for fresh soil. The SOC content was determined through acidification with 1 M HCl (soil: HCl [m/v] = 1:2.5) [41, 42], and total nitrogen (TN) was measured by combustion analysis of 0.15-mm sieved air-dried soil with an elemental analyzer (Vario Macro cube, Elementar). Total phosphorus (TP) was determined by the acid solution Mo-Sb anti-spectrophotometry method [43]. Dissolved organic carbon (DOC) was extracted into water (soil: water [m/v] = 1:5) via shaking for 24 h at 30 °C and then determined by the Elementar TOC Analyzer (Vario TOC; Elementar) after filtering through a 0.45-µm membrane (Merck KGaA). The ammonium and nitrate contents were determined after extraction of 2 g air-dried soil into 10 mL of 2 M KCl (aq) and then shaking for 2 h and standing for 30 min, followed by filtration with a 0.45-µm membrane (Merck KGaA). The contents were then determined with a continuous flow analyzer (AA3; SEAL).

### DNA extraction and high-throughput Illumina sequencing

Total genome DNA was extracted from 0.5 g soil according to the kit instructions of the MOBIO PowerSoil DNA Isolation Kit (MOBIO Laboratories, Carlsbad, CA, USA). The concentration and quality of DNA were detected by Qubit 4.0 (Thermo Fisher Scientific, Waltham, USA) and NanoDrop 2000 (Thermo scientific, Wilmington, DE, USA), which was prepared for high-throughput Illumina sequencing and quantitative microbial element cycling (QMEC).

The primers U341F (5′-ACTCCTACGGGAGGCAGCAG-3′) and U806R (5′-GGACTACHVGGGTWTCTAAT-3′) in the V3V4 regions of 16S rRNA [44] were used for the amplification to characterize the bacterial community. PCR reactions were performed in a 50-μL reaction system, containing 25 μL 2x Premix Taq (Takara Biotechnology, Dalian Co. Ltd., China), 1 μL of each primer (10 μM), and 3 μL of the DNA template (20 ng μL^−1^). The PCR process was conducted as follows: 5 min at 94 °C for initialization; 30 cycles of 30-s denaturation at 94 °C, 30-s annealing at 52 °C, and 30-s extension at 72 °C; followed by 10-min final elongation at 72 °C. After mixing in equidensity ratios, the PCR products were purified for sequencing on the Illumina Nova6000 platform (Guangdong Magigene Biotechnology Co., Ltd. Guangzhou, China). The obtained raw sequences were analyzed on the Galaxy pipeline (http://159.226.240.74:8080/) [45]. After removal of the primer sequences, the data were merged by FLASH, and the unqualified sequences were filtered out [45].

UPARSE with a 97% similarity threshold was used to remove chimera sequences and cluster the remaining sequences into operational taxonomic units (OTUs). The Greengenes 13.8 database [46] was used to classify OTU taxonomy. Total sequences per sample were rarefied to the minimum sum of OTUs among all samples after screening out the low-frequency OTUs for further analysis [47].

### Quantitative microbial element cycling (QMEC)

The high-throughput quantitative-PCR-based chip method QMEC was conducted to quantify 32 functional genes related to carbon cycling, using the 16S rRNA gene as the reference gene [35]. The relationships between genes and functions are shown in Table S2. The qPCR reaction and fluorescence signal detection were performed in SmartChip Real-Time PCR System (WaferGen Biosystems, USA), which automatically generates the amplification curve and dissolution curve. A non-template negative control was included for each run, and the samples were processed in triplicate. When the amplification efficiency was between 1.8 and 2.2, as well as satisfying the non-amplified negative control, the gene was included in further analysis. A threshold cycle (Ct) of 31 was used as the detection limit. The absolute abundance of genes was obtained based on the Ct value and the absolute quantitative information of the 16S rRNA gene [35].

### Statistical analysis

Indexes of bacterial alpha diversity including observed richness, Chao1, Shannon, and Pielou’s evenness were calculated in R 4.0.5. The differences of alpha diversity indexes, environmental factors, and abundance of functional genes with retreat years as the categorical variable were tested by one-way ANOVA and the Fisher’s least significant difference (LSD) method in the “agricolae” package. Pearson coefficients were calculated to examine the relationships among SOC, major bacterial phyla, and carbon-related functional gene groups. Principal coordinate analysis (PCoA) was performed based on the Bray-Curtis distance to evaluate the differences of bacterial community among the successional zones. Permutational multivariate analysis of variance (PERMANOVA) was carried out to verify the significance of the differences. These analyses, including heatmaps, were conducted in the “vegan” and “pheatmap” packages. And all the figures were plotted by the “ggplot2” packages.

For evaluating the impacts of retreat time and bacterial community on the change of SOC, the structural equation modeling (SEM) was conducted with AMOS 24 (AMOS IBM, USA). Bacterial abundance (copy number) could represent the bacterial biomass, and the gene abundance ratio between carbon fixation and carbon degradation (FD ratio) could imply the change of microbial carbon-related functional potentials. So they were incorporated into the SEM model as microbial position. Meanwhile, soil pH would affect the growth and functions of microorganisms which was also selected as explanatory variables. The priori model of SEM is shown in Fig. S1. When the model satisfy the Chi-square test (*χ*^2^, *p* > 0.05), and has a good fit with the root mean square error of approximation (RMSEA) less than 0.05, goodness-of-fit index (GFI) and normed fit index (NFI) larger than 0.90, the model would be accepted.

## Results

### Soil physicochemical properties

**Fig. 2:**
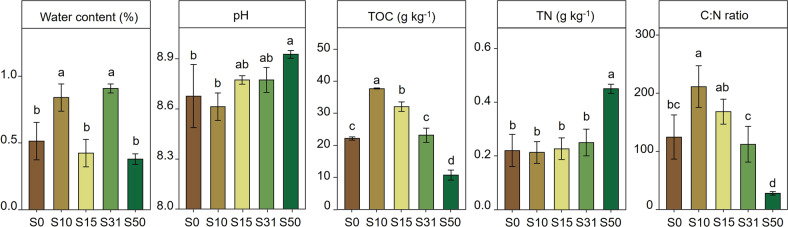
Soil physicochemical properties in the glacier foreland of Laohugou Glacier No. 12. The numbers in the five sampling sites represent the retreat years (S0, S10, S15, S31, and S50). Different letters indicate significant differences among sites (*p* < 0.05).

Soils were alkaline, with a slight increase in pH and with the highest pH value at S50 (8.93 ± 0.02) (Fig. 2 and Table S1). Under dry conditions with lower precipitation, the soil water content was in the range of 0.38~0.91% (Fig. 2 and Table S1). The SOC content showed a clear variation with soil age (Fig. 2). After reaching the maximum at S10 (37.80 ± 0.07 g kg^−1^), the SOC decreased significantly to about 10.77 g kg^−1^ at S50, which was 11.44 g kg^−1^ lower than that at S0 (Table S1). In contrast, the soil TN content significantly increased from 0.22 g kg^−1^ to 0.45 g kg^−1^ after 50 years’ receding, and the TP value was relatively stable, with a fluctuation range between 0.51 g kg^−1^ and 0.66 g kg^−1^ (Fig. 2 and Table S1). Similar to the SOC, the C:N and C:P ratios were the highest in S10, with 211.24 and 192.32, respectively, and then decreased to about 27.86 and 49.94 at S50, respectively (Table S1 and Fig. 2). Ammonium and nitrate contents decreased significantly, whose value at S50 was approximately half and one-sixth that of S0, respectively. In contrast, the value of DOC increased from 23.33 mg kg^−1^ (S0) to 37.85 mg kg^−1^ (S31) but then declined to 27.02 mg kg^−1^ at S50 (Table S1).

### Microbial carbon-related functional potentials

**Fig. 3:**
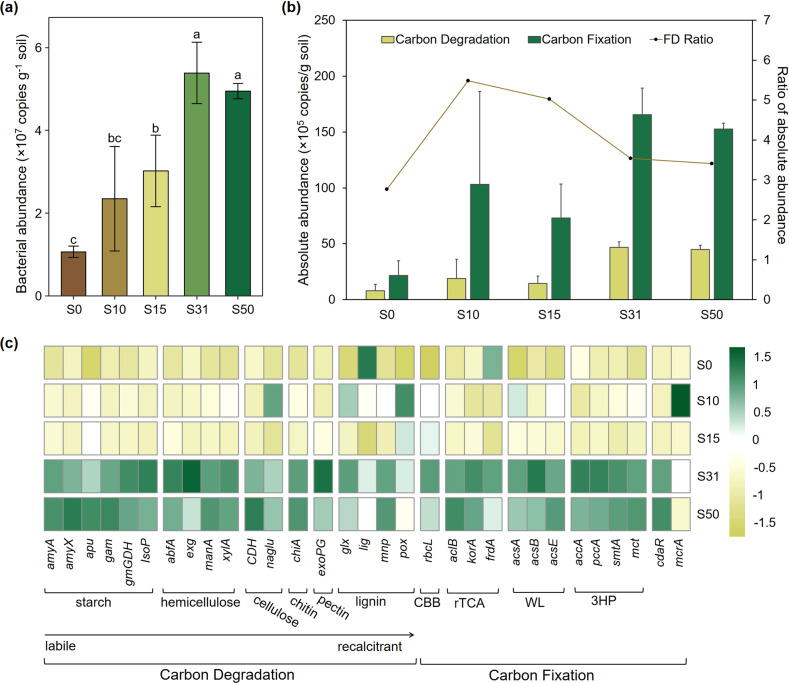
Absolute copy number of soil bacteria and functional genes in the glacier foreland of Laohugou Glacier No. 12. **a** Soil bacterial copy number; **b** absolute abundance and ratio (FD ratio) between carbon fixation and carbon degradation genes; **c** heatmap of absolute abundance of all functional genes. The numbers in the five sampling sites represent the retreat years (S0, S10, S15, S31, and S50). Different letters indicate significant differences among sites (*p* < 0.05). CBB, rTCA, WL, and 3HP represent the reductive pentose phosphate cycle, reductive tricarboxylic acid cycle, reductive acetyl-CoA pathway, and 3-hydroxypropionate bicycle carbon fixation pathways, respectively. Green shading indicates a higher absolute abundance, whereas yellow shading indicates a lower absolute abundance.

Although gene *exc* could not be found in all samples, a total of 31 functional genes were detected in all samples, including 18 genes related to carbon degradation and 13 genes associated with carbon fixation. Their copy numbers were calculated based on the abundance of bacteria which increased from 1.07 × 10^7^ copies g^−1^ soil to 4.95 × 10^7^copies g^−1^ soil during the 50 years of recession, with the peak value at S31 of 5.38 × 10^7^ copies g^−1^ soil (Fig. 3a and Table S3). Similarly, the abundances of almost all genes gradually increased with the peak value at S31, followed by a slight decrease at S50 (Table S3), except for the *naglu*, *glx*, *lig*, *pox*, *frdA*, *acsA*, and *mcrA* genes (Fig. 3c).

The abundance of functional genes responsible for carbon fixation increased about seven-fold, from 21.77 × 10^5^ copies g^−1^ soil (S0) to 152.79 × 10^5^ copies g^−1^ soil (S50). However, regardless of the successional zone, it was higher than that of carbon degradation, which increased 5.70 times, with 44.83 × 10^5^ copies g^−1^ soil at S50 (Fig. 3b and Table S3). The FD ratio peaked at 5.48 at S10 and then decreased to 3.41 at S50, which was not significantly different from the value of the initial stage (2.77) (Fig. 3b).

For comparing the different gene groups in carbon fixation genes, the relative abundances of each gene groups in carbon fixation genes were calculated as Table 1, which were based on the genes‘ functional attributes (Table S2) and their absolute abundance (Table S3). In every successional zone, most carbon fixation genes were attributed to the reductive acetyl-CoA pathway (WL cycle), which accounted for over 50% of all carbon fixation genes (S0: 1088.68 × 10^3^ copies g^−1^ soil, 50.01%; S50: 8832.08 × 10^3^ copies g^−1^ soil, 57.81%) (Table 1 and Table S3). The proportion of genes’ abundance which was related to the 3-hydroxypropionate bicycle (3HP cycle), the second largest carbon-fixation gene group, increased from 26.10% (S0: 568.14 × 10^3^ copies g^−1^ soil) to 30.41% (S50: 4645.86 × 10^3^ copies g^−1^ soil) (Table 1 and Table S3). The percentage of genes’ abundance associated with the reductive pentose phosphate (CBB) cycle also showed a slight increase from 3.37% at S0 to 5.81% at S50 (Table 1). Accounting for 20.52% of all carbon-fixation genes at S0, the genes involved in the reductive tricarboxylic acid (rTCA) cycle decreased to 5.96% at S50 (911.23 × 10^3^ copies g^−1^ soil) (Table 1 and Table S3).

**Table 1:** Relative proportion of carbon functional gene groups in the glacier foreland of Laohugou Glacier No. 12.

Microbial function	Gene groups	S0	S10	S15	S31	S50
Carbon fixation	CBB cycle	3.37%	6.90%	11.09%	6.83%	5.81%
	rTCA cycle	20.52%	3.65%	6.05%	5.71%	5.96%
	WL cycle	50.01%	70.50%	66.08%	59.81%	57.81%
	3HP cycle	26.10%	18.92%	16.78%	27.63%	30.41%
	Others	0.00%	0.04%	0.00%	0.02%	0.01%
Carbon degradation	Starch	21.09%	13.34%	17.33%	17.53%	19.34%
	Hemicellulose	46.03%	61.33%	62.27%	66.90%	64.70%
	Cellulose	2.40%	1.02%	1.16%	0.88%	1.09%
	Chitin	1.52%	1.60%	1.59%	1.46%	1.57%
	Pectin	0.00%	0.00%	0.19%	0.30%	0.19%
	Lignin	28.95%	22.71%	17.46%	12.93%	13.12%

Notes: Numbers for the five sampling sites represent the retreat years (S0, S10, S15, S31, and S50). The cycle of CBB, rTCA, WL, and 3HP represent the reductive pentose phosphate cycle, reductive tricarboxylic acid cycle, reductive acetyl-CoA pathway, and 3-hydroxypropionate bicycle carbon fixation pathways, respectively.

Similarly, the relative abundance of carbon degradation gene groups could be obtained as Table 1. The genes responsible for hemicellulose degradation were the most prevalent among all genes of carbon degradation, regardless of successional zone (Table 1). Proportion of hemicellulose degradation genes’ abundance to total carbon degradation genes’ abundance increased from 46.03% (S0: 361.74 × 10^3^ copies g^−1^ soil) to 64.70% (S50: 2900.58 × 10^3^ copies g^−1^ soil). Genes related to lignin and starch degradation were also dominant in carbon degradation genes, whose proportion decreased from 50.05% (S0: lignin: 28.95%, starch: 21.09%) to 32.46% (S50: lignin: 13.12%, starch: 19.34%) (Table 1). Moreover, the absolute abundance of genes involved in starch degradation (867.02 × 10^3^ copies g^−1^ soil, 19.34%) at S50 was higher than that of those involved in lignin degradation (588.09 × 10^3^ copies g^−1^ soil, 13.12%) (Table 1 and Table S3).

### Microbial community composition

**Fig. 4:**
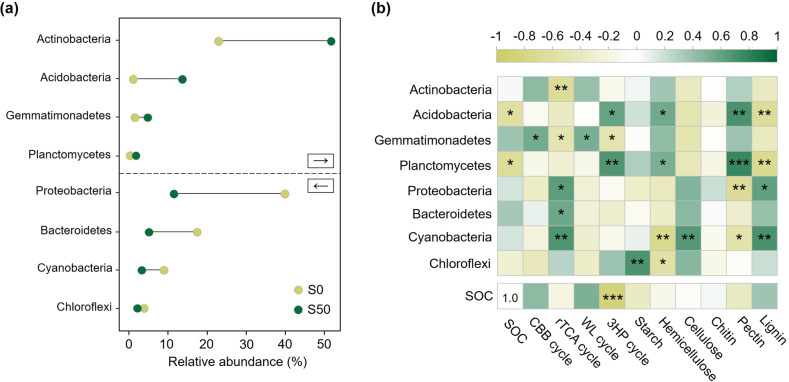
Relative abundance of major bacterial phyla and the relationships among retreat time, bacterial phyla, and functional gene groups in the glacier foreland of Laohugou Glacier No. 12. **a** Change of relative abundance of major bacterial phyla during 50 years of receding; (**b**) Pearson coefficients among soil organic carbon (SOC), relative abundance of bacterial phyla, and proportion of functional gene groups in carbon degradation and carbon fixation. Numbers in the five sampling sites represent the retreat years (S0, S10, S15, S31, and S50). The cycle of CBB, rTCA, WL, and 3HP represent the reductive pentose phosphate cycle, reductive tricarboxylic acid cycle, reductive acetyl-CoA pathway, and 3-hydroxypropionate bicycle carbon fixation pathways, respectively. Directions of arrows indicate the increase (pointing to the right) and decrease (pointing to the left) of relative abundance. “*,” “**,” and “***” indicate the significant differences of *p* < 0.05, 0.01, and 0.001, respectively. Green shading indicates a higher correlation, whereas yellow shading indicates a lower correlation.

After filtering out the unqualified sequences, a total of 437,265 high-quality sequences in the V3V4 region of 16 S rRNA gene were obtained, and the corresponding rarefaction curve is shown in Fig. S2. These sequences were clustered into 7693 OTUs, including 35 phyla. In the five successional zones of Laohugou Glacier No. 12 foreland, the dominant phyla were Actinobacteria and Proteobacteria (Fig. 4a). The relative abundance of Proteobacteria decreased remarkably from 39.64% at S0 to 12.32% at S50 (Fig. 4a), and the relative abundance of Bacteroidetes decreased by 12.64%. The abundance of Cyanobacteria continually decreased to 2.05% at S31 from 8.69% (S0), later showing a slight increase to 4.83% at S50 (Fig. 4a and Fig. S3). Chloroflexi showed a volatile decrease along the chronosequence from 3.64% (S50) to 1.50% (S0) (Fig. 4a and Fig. S3). In contrast, Acidobacteria, with a significant upward trend, comprised 13.41% of the bacterial community at S50, whereas it accounted for only 0.84% at S0. Despite initially showing an unstable increase with a slight decline at S31, Actinobacteria became the largest group with a relative abundance of 51.51% at S50 (Fig. 4a and Fig. S3).

### Diversity of bacterial community

In general, the alpha diversity of bacterial community showed an increasing trend (Fig. S4). The indexes, such as the observed richness, Chao1, Shannon index, and Pielou’s evenness, were similar in the first 15 years of receding, followed by a significant increase at S31 and S50. In contrast, the beta diversity of the bacterial community was significantly different among the five successional sites, as replicates of each sample clustered together and samples for each of the five sites were separated (Fig. S5). The first and second principal components of the PCoA explained 36.60% and 27.25% of the variation in the bacterial community, respectively. The PERMANOVA test also verified the significant difference with an *F* value of 6.71 and a *p* value of less than 0.001.

### Relationships between edaphic factor, bacterial community, and SOC

Based on the change of major bacterial phyla (Fig. 4a) and carbon-related functional genes (Table 1), their relationships with SOC were analyzed as Fig. 4b. The relative abundances of Acidobacteria and Planctomycetes were significantly negatively correlated with SOC as well as the relative proportion of genes involved in 3HP cycles. Acidobacteria had a significant positive correlation with the relative proportions of genes involved in the hemicellulose degradation, pectin degradation, and 3HP cycles, but were significantly negatively correlated with the relative proportion of genes involved in lignin degradation (Fig. 4b). Proteobacteria had a negative correlation with the relative proportions of genes associated with pectin degradation but a significant positive correlation with the relative proportions of genes associated with lignin degradation and the rTCA cycle. The relationships of Bacteroidetes were similar to those for Proteobacteria. With a weak correlation with SOC, Actinobacteria and Gemmatimonadetes were positively correlated with genes involved in the CBB and WL cycles but significantly negatively correlated with those of the rTCA cycle.

**Fig. 5:**
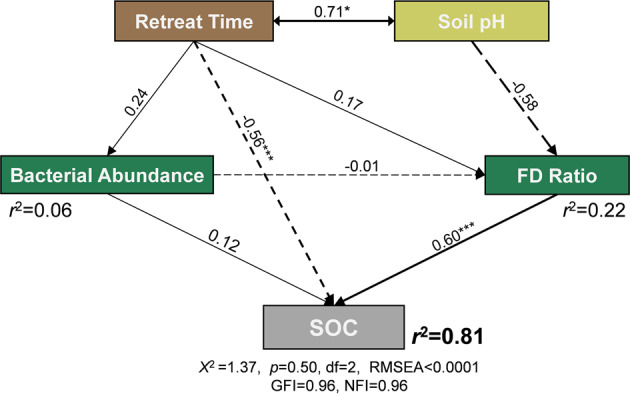
Structural equation modeling of the impacts of environmental factors and bacterial community on soil organic carbon (SOC) along the Laohugou Glacier No. 12 foreland. SOC and FD ratio denote soil organic carbon and gene abundance ratio between carbon fixation and carbon degradation. Numbers adjacent to arrows are standardized path coefficients. Arrow width is proportional to the strength of the path coefficients. Significance levels of each path are *p < 0.05, **p < 0.01, and ***p < 0.001. Dashed and solid lines indicate the negative and positive effects, respectively. The *r*^*2*^ values positioned next to each *r*esponse variable in the model denote the proportion of variance explained.

SEM was performed to quantify the impacts of retreat time, soil pH, bacterial abundance, and functional potentials on the change of SOC (Fig. 5). The SEM model could explain 81% of variations in SOC along the soil chronosequence, with a good fit of the *χ*^2^ test, RMSEA (less than 0.0001), GFI (0.96), and NFI (0.96). Direct positive effects of FD ratio on SOC content were observed (0.60), and without any observed indirect effects (Fig. S6). Similarly, bacterial abundance mainly had a direct effect (0.12) on SOC content, with indirect effects less than −0.001. In comparison, the retreat time and soil pH had negative total effects on SOC content. With indirect effects of 0.13, the direct effect of retreat time on SOC content was −0.56. Soil pH did not directly impact the change of SOC, but with indirect effects of −0.35 (Fig. S6).

## Discussion

### Carbon loss in the continental glacier foreland

During the 50 years of succession, SOC in the continental glacier foreland of Laohugou Glacier No. 12 increased and then declined, giving an overall decrease (Fig. 2). This contrasts with the continuous increase tendency that is generally observed in others glacier forelands [7]. After exposure for 31 years, SOC contents in the foreland of Urumqi Glacier No. 1 (sub-continental glacier) significantly increased from 2.93 g kg^−1^ to 6.16 g kg^−1^ [48]. Under the rapid successional rate, SOC contents in the foreland of Hailuogou Glacier (typical temperate glacier) increased from 0.10 g kg^−1^ to 1.49 g kg^−1^ after receding for 15 years [49]. There was also a significant increase of SOC in the forelands of Damma Glacier in Switzerland [50], Puca Glacier [51, 52], and Easton Glacier in America [53]. Thus, unlike other glacier types, soils in this typical continental glacier foreland are characterized by carbon loss, dry conditions, and low temperature. Taking the rates of glacier retreat and carbon loss of Laohugou Glacier No. 12 as a reference, the amount of carbon accumulation from all the continental glacier forelands in China could be overestimated (Supporting Information II). Special cases may arise due to the limited number of studied subjects, but it also implies the possibility of carbon loss in glacier foreland, which indicates a non-negligible feedback to global changing.

### Impacts of bacterial community and functional potentials on carbon

Nitrogen limitation would stimulate carbon synthesis to promote nitrogen accumulation [54], whereas when the C:N ratio reaches a very high value, carbon would be released [55]. In the initial stages, limitation of nitrogen and carbon would stimulate microorganisms to obtain nutrients, while the limited nitrogen was gradually alleviated along the soil chronosequence with lower C:N ratio, which also indicates the higher carbon requirement and the considerable carbon constraint [54, 55]. This carbon limitation would affect the growth of the bacterial community, which would impact the carbon cycle conversely [37, 56]. The SEM results confirmed the effects of bacterial community on the carbon cycle (Fig. 5). Bacterial abundance had direct positive effects on SOC content because the growth of bacteria improves the accumulation of microbial necromass in soil [57]. From the perspective of microbial functions, the FD ratio had a stronger direct effect on the SOC content. A lower FD ratio indicates the lower efficiency of carbon fixation and higher consumption of carbon during the 50-year recession, consistent with the change of SOC. This can explain the reduction of SOC because microbial carbon fixation and degradation are the important determinants of soil carbon contents in glacier forelands.

Based on the functional gene abundance, carbon fixation pathways were dominated by the WL, 3HP, and rTCA cycles in the initial stage, but in the later stages, only the first two types remained. Microorganisms can synthesize one molecule of oxalacetate from four molecules of carbon dioxide in one rTCA cycle [58], whereas only two molecules of carbon dioxide can be absorbed by microbial carbon fixation through one WL cycle or one 3HP cycle, which are more strict with the existence of oxygen [59]. This means that if the reduction of the rTCA cycle in carbon-fixation genes was replaced by the WL cycle and 3HP cycle, half of the fixed carbon could be reduced. After 50 years of succession of Laohugou Glacier No. 12 foreland, about 83% reduction of gene abundance involved in the rTCA cycle had been distributed in the WL and 3HP cycles, which indicates a considerable decrease in the amount of fixed carbon as well as the carbon fixation potentials. The relative proportion of genes related to the CBB cycle, which mainly depends on light energy [58, 60], did not significantly increase along the chronosequence (Table 1). Moreover, the relative abundance of phyla with autotrophic capacity such as Cyanobacteria, Chloroflexi, and some of Proteobacteria [60–62] decreased along the soil chronosequence (Fig. 4a). Accompanied by a shift of major phyla from Proteobacteria to Actinobacteria and Acidobacteria, the significant positive correlation between Proteobacteria and functional genes involved in the rTCA cycle also indicates a decreased carbon fixation capacity. Decreasing carbon fixation coefficient and fewer autotrophs would affect the fixed-carbon and may cause carbon loss.

A higher diversity of carbon compounds could provide more growth opportunities for microorganisms that would be beneficial to soil development and microbial diversity improvement [63, 64]. During the 50 years of glacier recession, carbon degradation genes were increasingly attributed to hemicellulose degradation (Table 1), consistent with the increasing abundance of Actinobacteria, which are known to degrade hemicellulose [65, 66]. Correspondingly, the proportion of other gene groups decreased, such as lignin, starch, lignin, and cellulose. This not only illustrates the increasing microbial hemicellulose degradation potentials but might also indicate a decrease in the diversity of carbon compounds in soils. Moreover, the change in microbial life strategy could also indicate the nutrient availability, for example, more K-strategists indicates lower carbon availability [67, 68]. Relative abundances of Actinobacteria and Acidobacteria (Fig. 4a), which are typical K-strategists with slower growth rate and higher competitive capacity [69, 70], increased along the chronosequence. In contrast, the relative abundances of *r*-strategists, Proteobacteria, and Bacteroidetes decreased (Fig. 4a) as these bacteria could quickly respond with high growth rates when resources become available [69]. The shift of the bacterial community from *r*-strategists to K-strategists also indicates the lower available carbon resources, which requires microorganisms to have a higher survival ability. More importantly, the increasing bacterial copy number along the soil chronosequence indicates an increasing microbial biomass [71]. This suggests a higher carbon requirement and strengthened respiration. The reduction in the available carbon resource and increasingly intense competition might explain the decline of bacterial community diversity in the final recession stages (Fig. S4). This also illustrates that microbial functional potentials can respond rapidly to environmental changes [34].

In summary, SOC decreases with the phenomenon of carbon loss along a continental glacier foreland (Laohugou Glacier No. 12), which is different from the relationships previously shown for other types of glacier forelands. An increase in bacterial copy number indicates an increase in carbon release from respiration and a higher microbial carbon requirement. Furthermore, the simplified carbon degradation gene groups and the bacterial community shift from *r*-strategists to K-strategists indicate the lower carbon availability. More importantly, a decrease of carbon fixation genes involved in the rTCA cycle as well as the relative abundances of phyla with autotrophic capacity show lower carbon-fixation efficiency and inadequate fixed-carbon replenishment. The combined influences of these factors partially contribute to the carbon loss in glacier foreland of Laohugou Glacier No. 12, which is considerably different from those previously shown for temperate glaciers and sub-continental glaciers. This carbon loss indicates a positive feedback of continental glacier forelands to climate warming, and taking this into account in future research could support the more accurate prediction of carbon storage in glacier forelands.

## Supplementary information

Supplemental Material

## Data Availability

The sequencing data had been uploaded on the National Center for Biotechnology Information Sequence Read Archive with the accession number PRJNA796111.
