# Supplementary material for: Unexpected high carbon losses in a continental glacier foreland on the Tibetan Plateau
Source: ISME Commun. 2022 Aug 9;2:68. doi: 10.1038/s43705-022-00148-x (PMC9723710; doi:10.1038/s43705-022-00148-x)
Supplement: Supplementary file 1 — Supplemental Material [file 43705_2022_148_MOESM1_ESM.docx]

**Supplemental Material**

**Title:** Unexpected high carbon losses in a continental glacier foreland on the Tibetan Plateau

**Running title:** carbon loss in a continental glacier foreland

Jiejie Zhang^1,2,3^, Anzhou Ma^1,4*^, Hanchang Zhou^1,4^, Xianke Chen^1,2,3^, Xiaorong Zhou^1,4^, Guohua Liu^1,4^, Xuliang Zhuang^1,4,5^, Xiang Qin^6^, Anders Priemé^7,8^, Guoqiang Zhuang^1,4*^

^1^Research Centre for Eco-Environmental Sciences, Chinese Academy of Sciences, Beijing, 100085, China

^2^Sino-Danish College of University of Chinese Academy of Sciences, Beijing, 101400, China

^3^Sino-Danish Center for Education and Research, Beijing, 101400, China

^4^College of Resources and Environment, University of Chinese Academy of Sciences, Beijing, 100049, China

^5^Institute of Tibetan Plateau Research, Chinese Academy of Sciences, Beijing, 100101, China

^6^Qilian Shan Station of Glaciology and Eco-environment, State Key Laboratory of Cryospheric Science, Northwest Institute of Eco-environment and Resources, Chinese Academy of Sciences, Lanzhou, 730000, China

^7^Department of Biology, University of Copenhagen, Copenhagen, DK-2100, Denmark

^8^Center for Permafrost, University of Copenhagen, Copenhagen, DK-1350, Denmark

**Summary Information**

Supporting information I: Table S1-S3, Figures S1-S6

Supporting information II

Reference

**Supporting Information I**

**Table S1 Soil physicochemical properties in Laohugou Glacier No. 12 foreland (mean ± SD, n=3)**

| **Sampling sites** | **S0** | **S10** | **S15** | **S31** | **S50** |
| --- | --- | --- | --- | --- | --- |
| Distance from ice tongue (m) | 0 | 128.14 | 198.41 | 320.86 | 389.21 |
| pH | 8.68±0.19 b | 8.61±0.08 b | 8.77±0.03 ab | 8.77±0.08 ab | 8.93±0.02 a |
| WC (%) | 0.51±0.14 b | 0.84±0.10 a | 0.42±0.10 b | 0.91±0.34 a | 0.38±0.39 b |
| SOC (g.kg^-1^) | 22.21±0.52 c | 37.80±0.07 a | 32.16±1.51 b | 23.22±2.24 c | 10.77±1.54 d |
| DOC (mg.kg^-1^) | 23.33±2.35 c | 23.47±1.23 c | 22.68±2.31 c | 37.85±1.58 a | 27.02±1.05 b |
| TN (g.kg^-1^) | 0.22±0.06 b | 0.21±0.04 b | 0.23±0.04 b | 0.25±0.05 b | 0.45±0.02 a |
| Ammonium (mg.kg^-1^) | 0.87±0.11 a | 0.74±0.21 a | 0.50±0.05 b | 0.51±0.11 b | 0.46±0.03 b |
| Nitrate (mg.kg^-1^) | 1.54±0.20 a | 1.19±0.06 b | 0.41±0.11 c | 0.29±0.04 c | 0.26±0.07 c |
| TP (g.kg^-1^) | 0.66±0.03 a | 0.51±0.04 b | 0.57±0.07 ab | 0.52±0.04 b | 0.56±0.09 ab |
| C:N ratio | 124.60±38.11 bc | 211.24±35.78 a | 168.05±21.49 ab | 112.09±30.71 c | 27.86±3.06 d |
| C:P ratio | 87.49±1.90 d | 192.32±14.17 a | 145.93±15.59 b | 114.68±6.63 c | 49.94±6.15 e |

Noted: Numbers in five sampling sites (S0, S10, S15, S31 and S50) represent the retreat years. Different letters indicate significant differences (*p* < 0.05). Abbreviations: WC, water content; SOC, soil organic carbon; DOC, dissolved organic carbon; TN, total nitrogen; TP, total phosphorus; C:N ratio, stoichiometric ratio of C (SOC) and N (TN); C:P ratio, stoichiometric soil of C (SOC) and P (TP).

**Table S2 The relationships between functional genes and microbial functions**

| **Functional group** | **function** | **Gene name** |
| --- | --- | --- |
| Carbon degradation [1] | Starch | *amyA, apu, amyX, gam, gmGDH, IsoP* |
|  | hemicellulose | *abfA, exgchi, manA, xylA* |
|  | cellulose | *CDH, naglu* |
|  | chitin | *chiA, exc* |
|  | pectin | *exoPG* |
|  | lignin | *glx, lig, mnp, pox* |
| Carbon fixation | reductive pentose phosphate cycle (CBB cycle) | *rbcL* [2] |
|  | reductive tricarboxylic acid cycle (rTCA cycle) | *aclB* [3]*, korA* [4]*,*  *frdA* [5] |
|  | reductive acetyl-CoA pathway (WL cycle) | *acsA, acsB, acsE* [6] |
|  | 3-hydroxypropionate bicycle (3HP cycle) | *accA, pccA, smtA, mct* [7] |
|  | others | *mcrA* [8] |
|  |  | *cdaR* [1] |

**Table S3 Absolute abundances of functional genes in Laohugou Glacier No. 12 foreland (mean ± SD, n=3)**

| **Gene name** | **Gene abundance (copies ×10^3^ g^-1^ soil)** | | | | |
| --- | --- | --- | --- | --- | --- |
|  | **S0** | **S10** | **S15** | **S31** | **S50** |
| 16S rRNA gene | 10660.08 ± 1399.67 c | 23450.44 ± 12623.36 bc | 30202.60 ± 8614.90 b | 53845.11 ± 7414.49 a | 49457.03 ± 1860.81 a |
| *abfA* | 40.19 ± 44.7 b | 141.46 ± 160.61 b | 236.16 ± 140.12 b | 770.64 ± 39.42 a | 645.18 ± 127.1 a |
| *amyA* | 3.1 ± 1.49 b | 8.78 ± 5.8 b | 8.89 ± 4.17 b | 24.77 ± 4.66 a | 26.26 ± 4.32 a |
| *amyX* | 0 ± 0 a | 0 ± 0 a | 0 ± 0 a | 3.16 ± 2.7 a | 4.24 ± 6.02 a |
| *apu* | 0 ± 0 c | 10.08 ± 6.05 b | 12.25 ± 5.59 b | 16.28 ± 1.42 ab | 21.68 ± 5.35 a |
| *CDH* | 18.89 ± 19.27 b | 16.47 ± 17.12 b | 16.91 ± 5.97 b | 39.11 ± 7.28 ab | 46.36 ± 9.64 a |
| *chiA* | 11.94 ± 7.25 b | 30.22 ± 24.13 b | 23.14 ± 11.85 b | 68.25 ± 14.91 a | 70.39 ± 13.79 a |
| *exg* | 17.47 ± 12.34 c | 19.19 ± 16.15 c | 14.52 ± 8.14 c | 114.49 ± 24.61 a | 62.41 ± 11.68 b |
| *exoPG* | 0 ± 0 c | 0 ± 0 c | 2.7 ± 1.76 c | 13.82 ± 3 a | 8.55 ± 0.52 b |
| *gam* | 126.93 ± 178.78 b | 161.52 ± 114.99 b | 172.23 ± 93.48 b | 525.33 ± 48.14 a | 595.91 ± 73.58 a |
| *glx* | 25.15 ± 11.81 b | 110.74 ± 96.09 ab | 56.36 ± 22.3 ab | 129.94 ± 36.7 a | 105.88 ± 7.39 ab |
| *gmGDH* | 14.72 ± 6.22 b | 50.4 ± 43.65 b | 39.14 ± 17.68 b | 160.17 ± 11.45 a | 144.28 ± 11.16 a |
| *IsoP* | 21.03 ± 18.57 b | 20.45 ± 14.22 b | 19.38 ± 9.48 b | 90.39 ± 14.84 a | 74.65 ± 15.51 a |
| *lig* | 86.45 ± 94.33 a | 56.84 ± 55.32 a | 35.69 ± 20.42 a | 65.85 ± 10.7 a | 60.91 ± 3.25 a |
| *manA* | 121.08 ± 20.23 b | 352.34 ± 323.5 b | 275.45 ± 115.17 b | 890.66 ± 221.56 a | 922.27 ± 140.33 a |
| *mnp* | 115.95 ± 90.51 b | 257.75 ± 273.83 ab | 160.24 ± 78.38 b | 407.27 ± 9.06 a | 420.25 ± 47.41 a |
| *naglu* | 0 ± 0 b | 2.69 ± 0.54 a | 0 ± 0 b | 2.14 ± 0.51 a | 2.29 ± 0.2 a |
| *pox* | 0 ± 0 c | 2.24 ± 0.7 a | 1.54 ± 0.08 b | 1.5 ± 0.09 b | 1.05 ± 0.25 b |
| *xylA* | 183.01 ± 99.31 b | 641.67 ± 591.94 b | 378.96 ± 145.2 b | 1353.47 ± 140.15 a | 1270.73 ± 159.93 a |
| *accA* | 24.63 ± 23.62 b | 9.55 ± 7.5 b | 14.64 ± 4.52 b | 67.56 ± 12.13 a | 55.16 ± 10.79 a |
| *aclB* | 46.03 ± 33.69 b | 99.76 ± 67.48 b | 88.46 ± 51.83 b | 270.83 ± 104.59 a | 296.77 ± 79.67 a |
| *acsA* | 866.62 ± 525.01 b | 6458.34 ± 5044.09 a | 4252.47 ± 2206.6 ab | 8519.53 ± 1190.49 a | 7403.22 ± 304.68 a |
| *acsB* | 0 ± 0 d | 2.8 ± 2.11 cd | 4.47 ± 2.45 c | 20.86 ± 4 a | 14.84 ± 1.29 b |
| *acsE* | 222.06 ± 108.61 b | 816.16 ± 672.71 ab | 572.92 ± 247.27 b | 1356.13 ± 293.09 a | 1414.03 ± 76.08 a |
| *cdaR* | 0 ± 0 b | 0 ± 0 b | 0 ± 0 b | 1.4 ± 0.74 a | 1.54 ± 0.61 a |
| *frdA* | 84.91 ± 97.33 a | 18.46 ± 13.42 a | 13.13 ± 7.83 a | 91.01 ± 16.59 a | 63.95 ± 23.13 a |
| *korA* | 315.74 ± 247.94 ab | 258.26 ± 216.76 b | 340.6 ± 157.89 ab | 583.2 ± 126.86 a | 550.51 ± 46.73 ab |
| *mcrA* | 0 ± 0 b | 4.37 ± 1.51 a | 0 ± 0 b | 1.25 ± 0.75 b | 0 ± 0 b |
| *mct* | 295.98 ± 122.34 b | 1597.88 ± 1498.64 b | 949.3 ± 363.42 b | 3631.3 ± 517.25 a | 3785.4 ± 551.26 a |
| *pccA* | 23.08 ± 14.44 b | 46.53 ± 37.09 b | 49 ± 22.67 b | 254.74 ± 12.96 a | 212.13 ± 70.69 a |
| *rbcL* | 73.32 ± 42.52 b | 711.81 ± 555.44 a | 810.7 ± 263.46 a | 1130.83 ± 262.45 a | 888.24 ± 33.26 a |
| *smtA* | 224.45 ± 210.37 b | 298.55 ± 266.73 ab | 213.64 ± 101.93 b | 618.67 ± 195.93 a | 593.17 ± 65.54 a |

Noted: Numbers in five sampling sites (S0, S10, S15, S31 and S50) represent the retreat years. Different letters indicate significant differences (*p* < 0.05).

**Figure S1 A priori model of Structural equation modeling (SEM).** The impacts of retreat time, soil pH, bacterial abundance (copy number) and gene abundance ratio between carbon fixation and carbon degradation (FD ratio) on soil organic carbon (SOC).


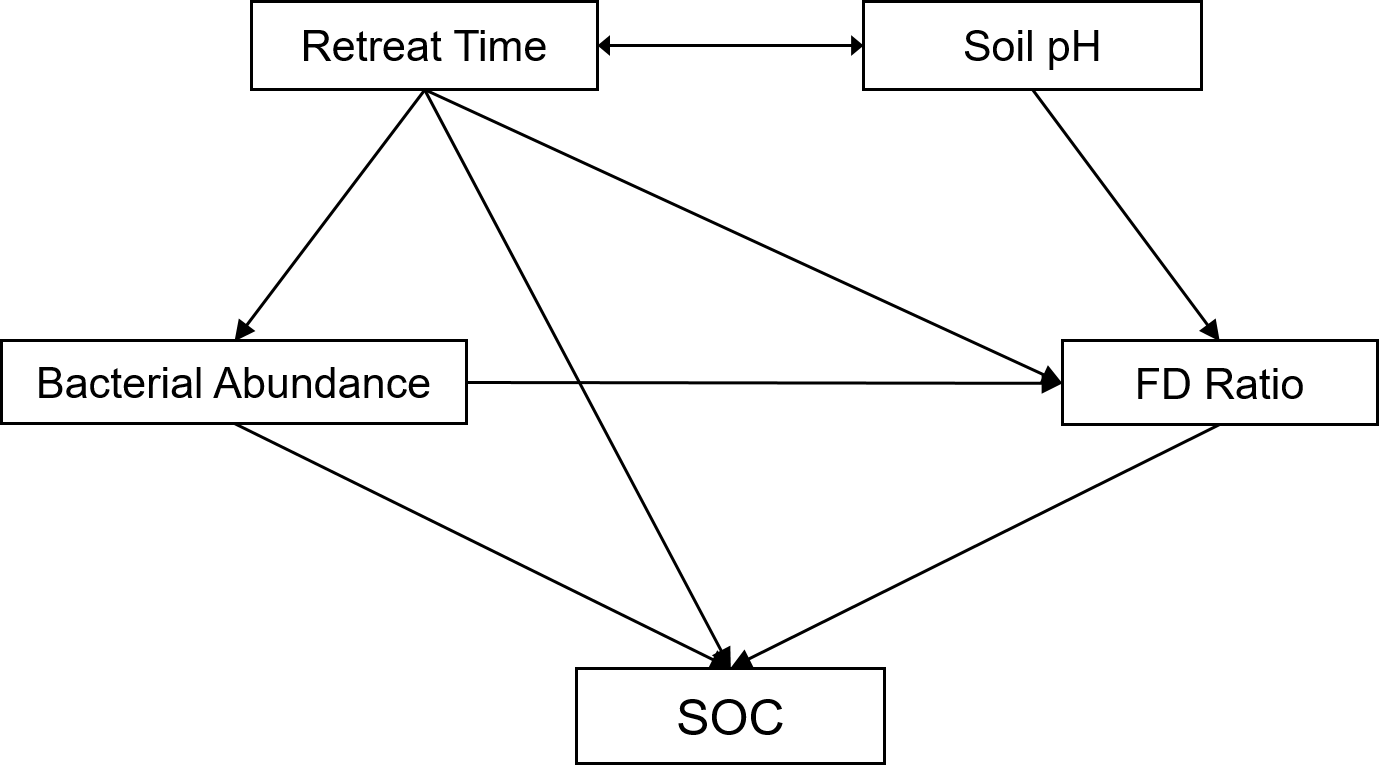


**Figure S2 Rarefaction curves for bacterial community of Laohugou Glacier No. 12 foreland.** Numbers in five sampling sites (S0, S10, S15, S31 and S50) represent the retreat years.


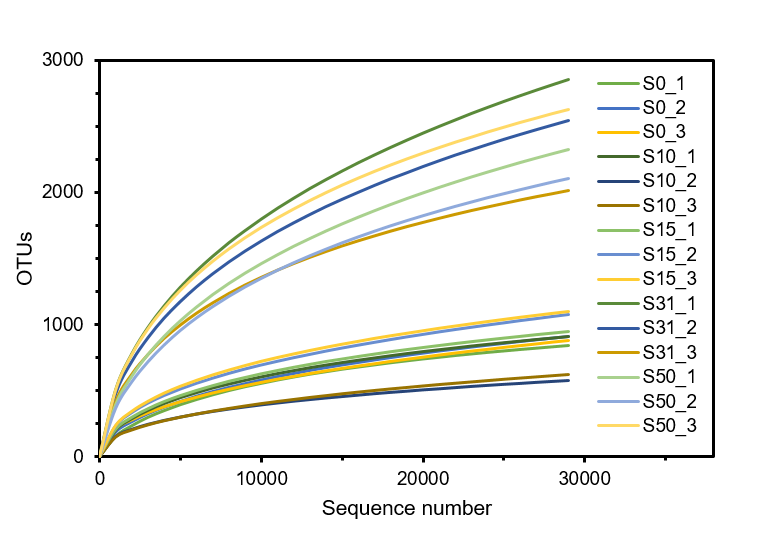


**Figure S3 Bacterial community composition in glacier foreland of Laohugou Glacier No. 12.** Numbers in five sampling sites (S0, S10, S15, S31 and S50) represent the retreat years.


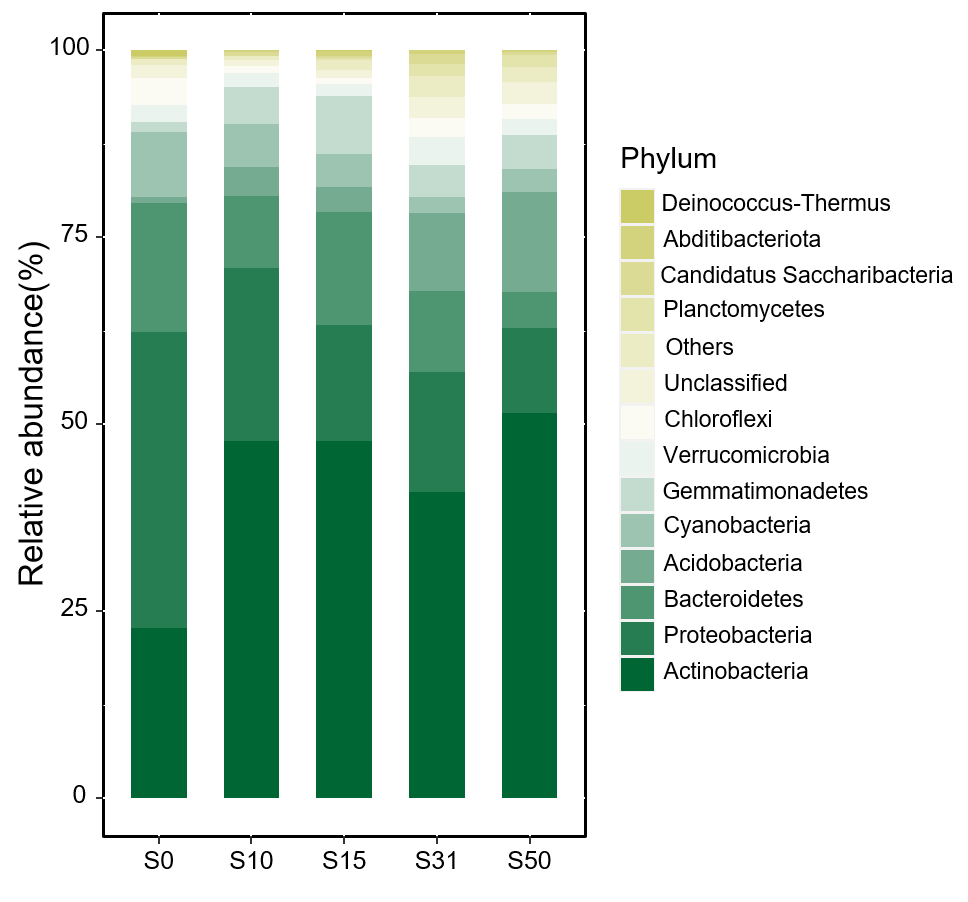


**Figure S4 Alpha diversity of bacterial community in glacier foreland of Laohugou Glacier No. 12.** Numbers in five sampling sites (S0, S10, S15, S31 and S50) represent the retreat years. Different letters indicate significant differences (*p* < 0.05).


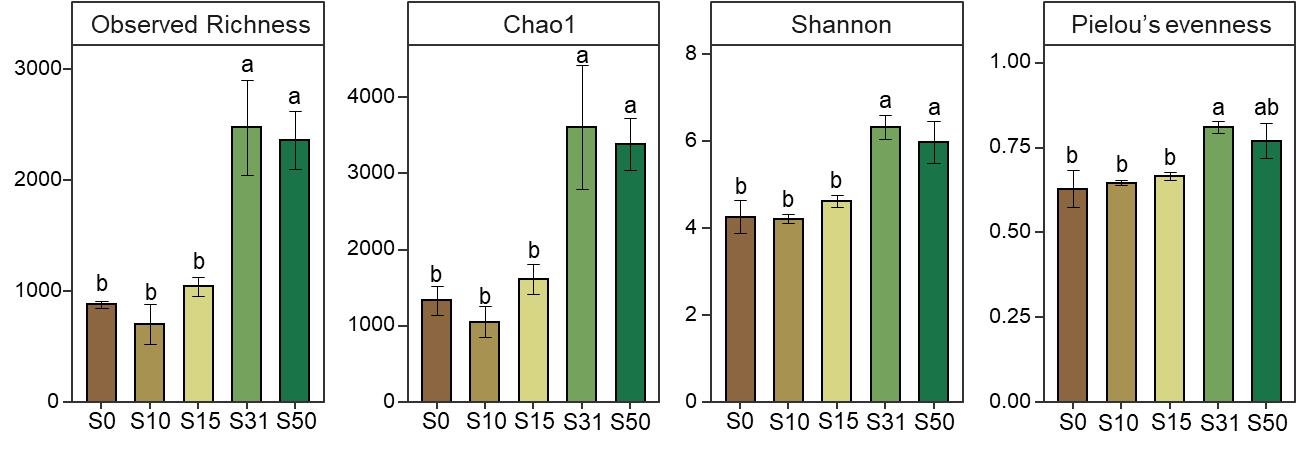


**Figure S5 Principal coordinate analysis (PCoA) analysis of bacterial community in glacier foreland of Laohugou Glacier No. 12.** Numbers in five sampling sites (S0, S10, S15, S31 and S50) represent the retreat years.


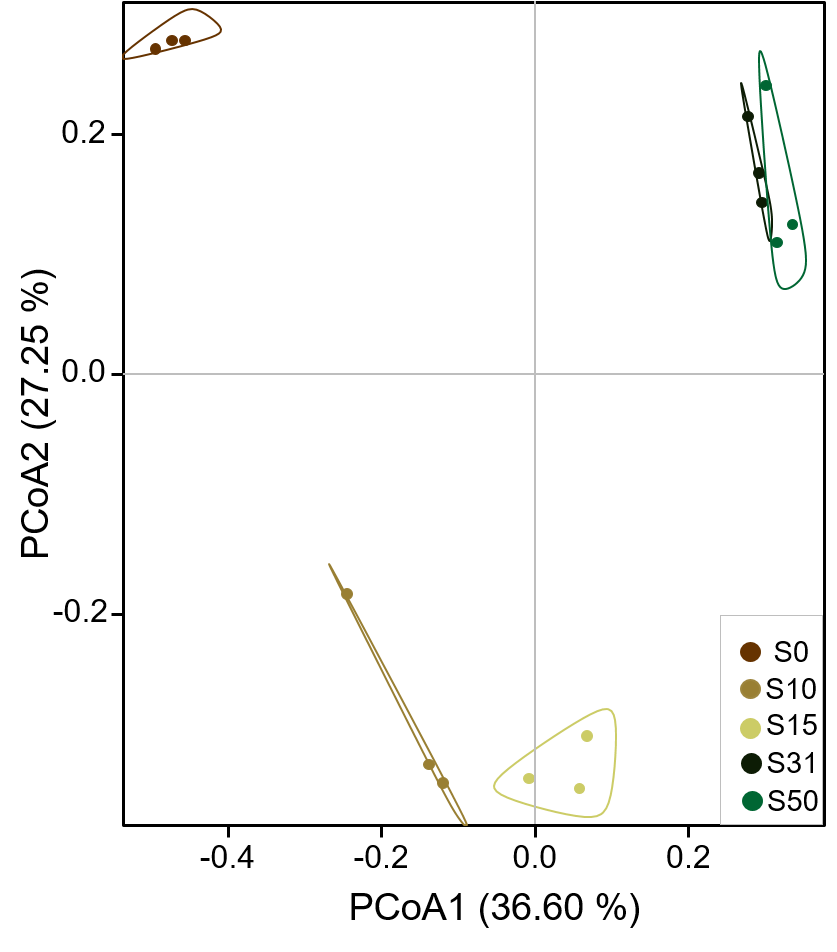


**Figure S6 Standardized total, direct and indirect effects of each explanatory variables on soil organic carbon (SOC) of structural equation modeling (SEM).** SOC and FD ratio denote soil organic carbon and gene abundance ratio between carbon fixation and carbon degradation.

**
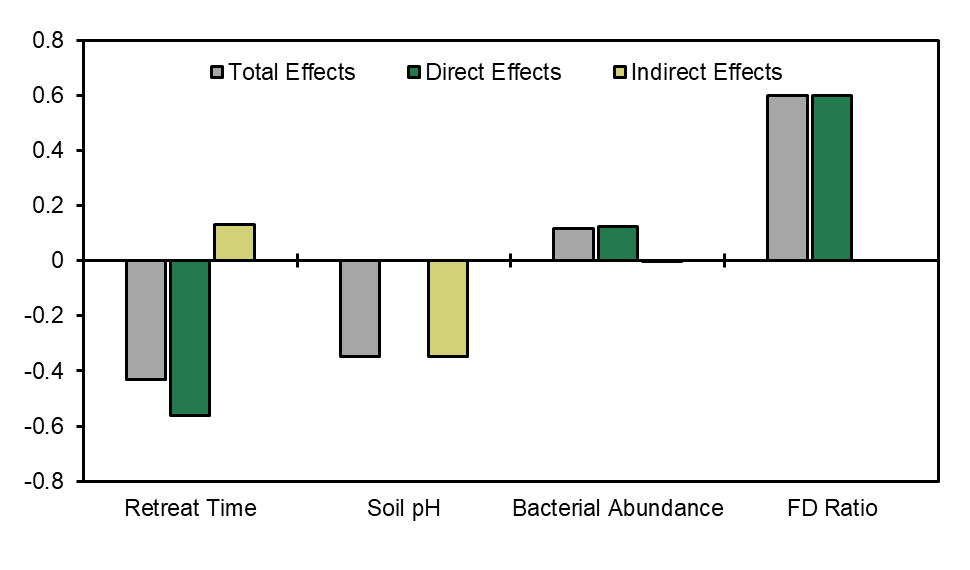
**

**Supporting Information II**

During 50 years’ succession, SOC decreased from 22.21 g.kg^-1^ to 10.77 g kg^-1^, which indicated the carbon loss rate was 0.2288 g kg^-1^ year^-1^, calculated as followed.

$$v_{{\_C}_{loss}}=\frac{22.21-10.77}{50}g.{kg}^{-1}.{year}^{-1}=0.2288 g.{kg}^{-1}.{year}^{-1}$$

All the continental glaciers cover 19000km^2^, occupying one third of glacier area in China. While, with an area of 21.03 km^2^, area of Laohugou Glacier No. 12 had decreased by 1.54 km^2^ in 1957 ~ 2015 [9]. So we hypothesized that other continental glaciers were similar, then the retreat area per year of continental glaciers in China would be calculate as followed.

$$v_{{\_area}_{loss}}=\frac{1.54}{(2015-1957)}\times\frac{19000}{21.03}{km}^{2}.{year}^{-1}=23.9887 {km}^{2}.{year}^{-1}=23.9887 \times{10}^{6} m^{2}.{year}^{-1}$$

Based on the bulk density ($\rho$=1.32 g cm^-3^=1.32 × 10^3^ kg m^-3^ ) [10] and depth of surface soil (0 ~ 10 cm, h=10 cm=0.01m), we could calculated the carbon loss from continental glacier due to the receding.

$$m_{{\_C}_{loss}}=v_{{\_C}_{loss}}\times\rho\times h\times v_{{\_area}_{loss}}=0.2288\times1.32 \times{10}^{3}\times0.01\times23.9887 \times{10}^{6} g.{year}^{-1}=7.245\times{10}^{7}g.{year}^{-1}=72.45 t.{year}^{-1}$$

That means due to the global warming and glacier retreat, there might have 72.45 ton carbon would be release every year in Chinese continental glacier forelands.

**References**

1. Zheng BX, Zhu YG, Sardans J, Peñuelas J, Su JQ. QMEC: a tool for high-throughput quantitative assessment of microbial functional potential in C, N, P, and S biogeochemical cycling. Sci China (Life Sciences). 2018; 61:1451–1462.

2. Xu HH, Tabita FR. Ribulose-1,5-bisphosphate carboxylase/oxygenase gene expression and diversity of Lake Erie planktonic microorganisms. Appl Environ Microbiol. 1996; 62:1913–1921.

3. Campbell BJ, Cary SC. Abundance of reverse tricarboxylic acid cycle genes in free-living microorganisms at deep-sea hydrothermal vents. Environ Microbiol. 2004; 70:6282–6289.

4. Yun N, Yamamoto M, Arai H, Ishii M, Igarashi Y. A novel five-subunit-type 2-oxoglutalate: ferredoxin oxidoreductases from *Hydrogenobacter thermophilus* TK-6. Biochem Biophys Res Commun. 2002; 292:280–286.

5. Barak R, Giebel I, Eisenbach M. The specificity of fumarate as a switching factor of the bacterial flagellar motor. Mol Microbiol. 1996; 19:139–144.

6. Pierce E, Xie G, Barabote RD, Saunders E, Han CS, Detter JC, et al. The complete genome sequence of *Moorella thermoacetica* (f. *Clostridium thermoaceticum*). Environ Microbiol. 2008; 10:2550–2573.

7. Shih PM, Ward LM, Fischer WW. Evolution of the 3-hydroxypropionate bicycle and recent transfer of anoxygenic photosynthesis into the Chloroflexi. Proc Natl Acad Sci USA. 2017; 114:10749–10754.

8. Liang B, Wang LY, Mbadinga SM, Liu JF, Yang SZ, Gu JD, et al. *Anaerolineaceae* and *Methanosaeta* turned to be the dominant microorganisms in alkanes-dependent methanogenic culture after long-term of incubation. AMB Express. 2015; 5:1–13.

9. Liu YS, Qin X, Chen JZ, Li ZL, Wang J, Du WT, et al. Variations of Laohugou Glacier No.12 in the western Qilian Mountains, China, from 1957 to 2015. J Mt Sci. 2018; 15:25–32.

10. Chai H, He NP. Evaluation of soil bulk density in Chinese terrestrial ecosystems for determination of soil carbon storage on a regional scale. Acta Ecol Sin. 2016; 36:3903–3910.
